# Protein-Protein Interaction Disruptors of the YAP/TAZ-TEAD Transcriptional Complex

**DOI:** 10.3390/molecules25246001

**Published:** 2020-12-18

**Authors:** Ajaybabu V. Pobbati, Brian P. Rubin

**Affiliations:** 1 Department of Cancer Biology, Lerner Research Institute, Cleveland Clinic Foundation, Cleveland, OH 44195, USA; 2 Robert J. Tomsich Pathology and Laboratory Medicine Institute, Cleveland Clinic Foundation, Cleveland, OH 44195, USA

**Keywords:** TEAD, YAP, TAZ, protein-protein interaction disruptors, PPID, Hippo pathway, transcription

## Abstract

The identification of protein-protein interaction disruptors (PPIDs) that disrupt the YAP/TAZ-TEAD interaction has gained considerable momentum. Several studies have shown that YAP/TAZ are no longer oncogenic when their interaction with the TEAD family of transcription factors is disrupted. The transcriptional co-regulator YAP (its homolog TAZ) interact with the surface pockets of TEADs. Peptidomimetic modalities like cystine-dense peptides and YAP cyclic and linear peptides exploit surface pockets (interface 2 and interface 3) on TEADs and function as PPIDs. The TEAD surface might pose a challenge for generating an effective small molecule PPID. Interestingly, TEADs also have a central pocket that is distinct from the surface pockets, and which small molecules leverage exclusively to disrupt the YAP/TAZ-TEAD interaction (allosteric PPIDs). Although small molecules that occupy the central pocket belong to diverse classes, they display certain common features. They are flexible, which allows them to adopt a palmitate-like conformation, and they have a predominant hydrophobic portion that contacts several hydrophobic residues and a small hydrophilic portion that faces the central pocket opening. Despite such progress, more selective PPIDs that also display favorable pharmacokinetic properties and show tolerable toxicity profiles are required to evaluate the feasibility of using these PPIDs for cancer therapy.

## 1. Introduction

Transcription factors are defined by their ability to access DNA through distinct DNA-binding domains (DBDs) [1]. DBDs recognize specific sequences that are frequently present at the promoter and enhancer regions of genes. In addition to DBDs, transcription factors possess transactivation domains that serve as a protein-protein interaction module allowing for the recruitment of co-activators, co-repressors, general transcription machinery or chromatin-modifying complexes. General transcription machinery is sufficient for the basal expression of all genes. However, to elicit profound and regulated changes in gene expression, the general transcription machinery needs to interact with sequence-specific transcription factors. As transcription factors control the expression of multiple genes, modulating their activity using pharmacological tools, such as small molecules, can profoundly change the behavior of cells to alter the disease course [2]. However, it is a treacherous road because more often than not, combating disease requires selective interventions, and predicting selectivity is difficult when modulating the activity of a single transcription factor can alter the expression of multiple target genes. Yet, some transcription factors, like the nuclear receptors, have emerged as ideal druggable candidates because they are not only disease drivers, but also possess targetable ligand-binding domains that bind to naturally occurring lipid-soluble ligands [3]. Ligand binding stimulates transcription and by intervening with this process, drugs like tamoxifen and enzalutamide are now used to block estrogen, and androgen-dependent transcription, respectively [3].

Mechanistically, not all transcription factors switch on transcription through binding to ligands. For these transcription factors, their activity can be modulated through intervening with their ability to bind to either DNA or other proteins. However, targeting these interactions, especially using small molecules, is anything but trivial. That said, tremendous progress has been made in the identification of transcription factor protein-protein interaction disruptors (PPIDs). Nutlins are exemplary in this regard—they potently disrupt the interaction of the transcription factor p53 with the ubiquitin ligase MDM2. Other prominent examples of transcription factor PPIDs disrupt Nrf2-Keap1 and HIF-pVHL interactions and Stat3 dimerization [4].

**Figure 1:**
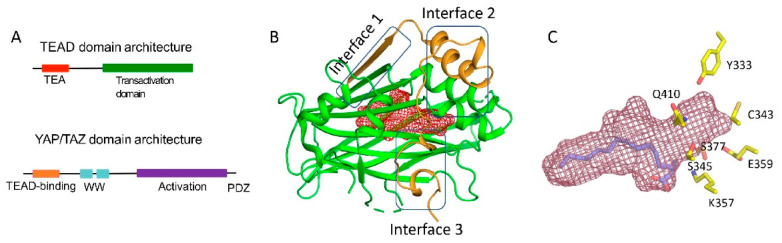
Domain architecture and structures of YAP/TAZ and TEAD (**A**) Domain architecture of YAP and TEAD (**B**) Ribbon diagram of the core complex structure of the YAP-TEAD complex (PDB ID: 3KYS). N-terminus of YAP interacts with the transactivation domain of TEAD by forming three interfaces. The central pocket is shown as red mesh. (**C**) Central pocket (red) and the orientation of the palmitate in the pocket is shown. The conserved polar/charged TEAD2 residues lining the central pocket is shown as yellow sticks.

PPIDs also act on the TEAD (TEA domain) family of transcription factors, which is the focus of this review [5,6,7]. They disrupt the interaction of TEADs with partners YAP (yes-associated protein) and TAZ (transcriptional co-activator with PDZ-binding motif). YAP and TAZ are paralogs and potent transcriptional co-regulators. Physiologically, the activity of YAP/TAZ is restricted by the Hippo signaling pathway and mechanical cues, such as cell geometry and stiffness [8,9]. A YAP-TEAD or a TAZ-TEAD complex initiates oncogenic transcriptional programs that are pivotal in tumor development, metastasis, and drug resistance [10]. YAP/TAZ must pair with TEADs to regulate transcription. The TEAD DBD allows YAP/TAZ to gain access to regulatory regions of genes and subsequently stimulate transcription through their activation domains (Figure 1A). Therefore, PPIDs that disrupt this interaction silence YAP/TAZ-TEAD transcription.

Being natively unfolded [11], it remains a challenge to target YAP and TAZ directly. To date, only the drug verteporfin has been shown to bind to YAP and act as a PPID [12]. On the other hand, several modalities target TEADs, and we highlight them in this review. We have previously referred to molecules that directly bind to YAP/TAZ or TEAD as group II modalities to distinguish them from group I drugs that act upstream and indirectly inhibit YAP/TAZ activity and, from group III drugs that reduce the effect of YAP/TAZ by inhibiting one or more of the downstream transcriptional targets [6]. Among the group II modalities are the PPIDs that bind to pockets on the TEAD surface at the YAP/TAZ interface. Interestingly, TEADs also possess a druggable central pocket that is distinct from the YAP/TAZ-binding surface [13]. Small molecules have been shown to interact with this pocket and disrupt YAP/TAZ-TEAD interaction. We will refer to these as allosteric PPIDs in this review.

## 2. Structural Characterization of YAP-TEAD Interaction

The TEADs are a family of four conserved proteins (TEAD 1-4 in humans) that have similar domain architecture (Figure 1A) [14,15]. All TEADs have an N-terminal TEA domain that binds to DNA and adopts a homeodomain fold. At the C-terminus, all TEADs have a transactivation domain that binds to YAP/TAZ. TEADs differ largely at the linker that connects the TEA domain with the transactivation domain. It was known early on that an N-terminal motif of YAP interacts with the TEAD transactivation domain (Figure 1A,B). However, the determination of the crystal structures of TEADs in complex with YAP was a breakthrough as it gave a clear, detailed picture of the interaction [11,16,17]. The TEAD transactivation domain adopts an immunoglobulin-like β-sandwich fold; additionally, it has two helix-turn-helix motifs. The N-terminal motif of YAP wraps around TEAD extensively and forms three interfaces (Figure 1B). Interface 1 is an anti-parallel β-sheet where both YAP and TEAD contribute to a single β-strand. This interface contributes little to the affinity between YAP and TEAD. Although there are drugs that target higher-order oligomers of β-sheets such as the amyloid plaques formed in Alzheimer disease [18], a simple β-sheet as in interface 1 has poor ligand-binding ability. Interface 2 is formed when a helix of YAP/TAZ slots into a pocket formed by one of the helix-turn-helix motifs of TEAD (Figure 1B). Such a helix-pocket interaction commonly occurs between proteins and is usually mediated by leucine residues (the LxxLL motif) [19,20]. These three hydrophobic leucine residues that are oriented towards the pocket are the key contact residues. YAP and TAZ have a variant of this motif, they have L^65^xxL^68^F^69^ motif instead of LxxLL (the numbers correspond to human YAP residues). Peptides and small molecules have been shown to occupy the interface 2 pocket (Table 1). The YAP/TAZ helix on its own binds poorly to TEAD and interface 3 acts to increase its binding affinity. Interface 3 is the most crucial of the three interfaces [11,16,17]. At this interface, YAP adopts a three-dimensional structure resembling the Greek letter Ω (Figure 1B), and this conformation is generally referred to as the Ω-loop. Crucial for interface 3 interaction are three hydrophobic core residues—in human YAP, these are M^86^, L^91^, and F^95^—that fit into a pocket on the TEAD surface. A cation-π interaction formed between R^87^ and F^96^ YAP residues is also crucial and appears to stabilize the Ω-loop conformation. Appreciable progress has been made on the peptidomimetics front, linear and cyclic peptides have been designed to act as PPIDs by occupying the interface 3 pocket on TEAD (Table 1). Interface 3 TEAD residues that interact with YAP are highly conserved, therefore it is difficult to design modalities at this interface that display selective TEAD inhibition. That said, it is not clear whether there is a biological need for selective TEAD inhibitors. In the field, researchers tend to work more on YAP than TAZ, but as YAP and TAZ have similar primary sequences and structural features, PPIDs that disrupt the YAP-TEAD interaction should also have the ability to disrupt the TAZ-TEAD interaction.

Interestingly, Pobbati and colleagues identified a pocket with an appropriate geometry and hydrophobicity in the center of the transactivating domain of TEADs that is accessible to small molecules [13] (Figure 1B). This pocket is distinct from the TEAD surface pockets. One end of the pocket is solvent-exposed. However, it is blocked by the interface 1 β-strand of YAP that acts as a gate to ligand access. The other end of the central pocket extends into the domain interior. Palmitate is the natural ligand that binds to the central pocket [31,32]. In addition to the hydrophobic residues that line the TEAD central pocket, there are seven conserved charged/polar residues (Figure 1C). Remarkably, all seven residues are located at the solvent-exposed end of the pocket. Notably, several central pocket binders have been identified that, not only inhibit TEAD palmitoylation, but also disrupt YAP-TEAD interaction, thus, acting as allosteric PPIDs. Several other molecules bind the pocket but only act as palmitoylation inhibitors and not as allosteric PPIDs. Despite this, they inhibit TEAD activity and function (Table 2).

## 3. Targeting the Interface 2 Region of the YAP-TEAD Complex

When the YAP-TEAD structure was first determined, a peptidomimetic approach to develop a PPID that could disrupt the interaction by exploiting interface 2 appeared to be an obvious choice. Stapled peptides mimicking the YAP or TAZ helix at interface 2 can prevent the functional interaction between YAP/TAZ and TEAD. Stapled peptides use hydrocarbon staples to stabilize the α-helix that makes them protease-resistant and endows them with an ability to cross cell membranes [44]. There are no reports of PPIDs successfully designed using this methodology. Molecular dynamics simulations have shown that hydrocarbon stapling induced the interface 2 helix of YAP to adopt an α-helical conformation and also reduced the entropy penalty [45]. However, the helix of YAP/TAZ that forms interface 2 binds poorly to TEADs, which makes designing a potent YAP/TAZ stapled peptide difficult. One strategy to circumvent this issue is by using the VGLL helix (vestigial-like) proteins for generating stapled peptides. The VGLL protein (also called TDU) helix that forms interface 2, interacts with TEADs much more potently than the YAP helix, partly due to the presence of the VxxHF motif instead of the LxxLF motif [46,47]. Recently, a tertiary structure mimetic of VGLL4 was used as a PPID to disrupt the interaction between TEAD and VGLL4 [48]. VGLL proteins (VGLL1-4 in humans) are another class of TEAD-binding co-regulators that mimic YAP/TAZ by forming structurally similar interfaces [14,49,50]. As an alternate strategy, residues of YAP/VGLL interface 3 can be coupled to interface 2 as coupling interface 3 residues contributes to a 400-fold increase in potency [13]. The Super-TDU peptide has been used as a PPID to inhibit YAP-TEAD interaction by disrupting binding at both interface 2 and 3 [30]. Super-TDU is a fusion of the VGLL4 helix and YAP Ω-loop residues. The peptide has remarkable cell-penetrating abilities, and was able to inhibit cell proliferation in gastric cancer cell lines and in a gastric cancer mouse model.

**Figure 2:**
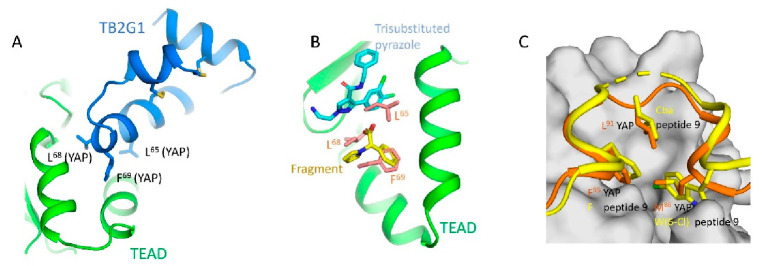
Modalities that occupy the surface pockets on TEADs (**A**) A model of the cystine-dense peptide (TB2G1) at the interface 2 pocket. The residues corresponding to the LxxLF motif of YAP is shown as sticks. (**B**) Crystal structures show that a fragment and the small molecule trisubstituted pyrazole occupy the interface 2 pocket on TEADs. Given the close proximity of the small molecule and the fragment, they could be linked to generate a potentially potent PPID. LxxLF residues of YAP is shown as salmon-colored sticks (**C**) Interface 3 region showing the omega-loop conformation of YAP (orange) (PDB ID: 3JUA) and peptide 9 (yellow) (PDB ID: 6Q36). The hydrophobic core residues in YAP and peptide 9 are shown as sticks and the interface 3 pocket on TEAD is shown in grey; Cba–cyclobutylalanine, W(6-Cl)–6-chlorotryptophan.

An optimized cystine-dense peptide (CDP) TB2G1 is one of the remarkable examples of a PPID that is able to bind to TEAD at interface 2 in the sub-nanomolar range (Figure 2A) [21]. As the name suggests, CDPs are small peptides of 10–80 amino acids that are rich in cysteines that use disulfide bridges to stabilize the protein conformation. Crook and colleagues extensively used the Rosetta modeler to identify the appropriate CDP scaffolds that would incorporate YAP residues. They also predicted the amino acid substitutions in YAP that would improve binding to TEADs. They extensively tested several of these CDPs using a mammalian surface display platform and identified TB1G1 as a stable and optimal CDP that retained an in vitro potency to TEAD in the nanomolar range. They then improved TB1G1 binding through affinity maturation, identifying five mutations that would improve its binding to TEAD—the quintuple mutant is TB2G1. Both TB1G1 and TB2G1 retain the key LxxLF motif and can disrupt the YAP-TEAD interaction as shown in co-immunoprecipitation and competitive binding experiments. The disadvantage here is a poor cell-penetrating ability, and the authors propose that it could be improved by adding cell-penetrating motifs. Cell penetrating motifs have been added to other peptides, the tripartite peptides NLS18-TEAD and NLS23-TEAD have been designed with cell penetrating motifs, but they use a sequence derived from TEAD to interfere with the YAP-TEAD interaction [51].

Peptidomimetics but not small molecules optimally function as PPIDs by occupying the interface 2 pocket on TEADs. However, significant progress is being made in the identification of small molecules that exploit interface 2. A small molecule fragment has been identified to bind interface 2 [22]. Interestingly, a phenyl moiety of the fragment has been shown occupying interface 2, mimicking the interaction of the phenylalanine of the LxxLF motif (Figure 2B), but the fragment is not potent enough to act as a PPID.

Through X-ray crystallography, several tri-substituted pyrazoles have been convincingly shown to occupy the TEAD pocket that forms interface 2. Structures of five analogs are deposited in the RCSB Protein Data Bank (PDB IDs: 6S6J, 6S60, 6S64, 6S66 and 6S69). These compounds should potentially disrupt the N-terminal helical region of YAP/TAZ to prevent interaction with TEAD. They are located around the position occupied by the L^65^ residue of the L^65^xxL^68^F^69^ motif but do not extend further (Figure 2B).

## 4. PPIDs that Bind Interface 3

The most effective PPIDs could be generated by interfering with binding at interface 3 as this is the most important of the three YAP/TAZ interfaces. From a peptidomimetic standpoint, considerable progress has occurred. Two YAP cyclic peptides derived from interface 3 residues that have their Ω-loop conformations stabilized through optimized disulfide bonds have been shown in two studies to effectively disrupt YAP-TEAD interaction in vitro [23,24]. When optimized, peptide 17 and peptide 10 have a tremendous improvement in potency compared to native YAP peptides of similar length. From YAP’s three hydrophobic core residues on interface 3 (M^86^, L^91^, and F^95^), only the F^95^ is retained in both the peptides. The L^91^ residue is altered to norleucine (Nle) in peptide 17 and to 2-aminoheptanoic acid (Ahe) in peptide 10. Nle and Ahe are structurally similar, except for the additional methylene in Ahe. Additionally, in peptide 17, M^86^ is modified to meta-chloro phenylalanine Phe(3-Cl), which greatly improved the potency [23]. The disulfide bond in these peptides acts as a substitute for the cation-π interaction formed between the R^87^ and F^96^ YAP residues. A 15-mer linear peptide has also been found to disrupt YAP-TEAD interaction in the nanomolar range [25]. The TEAD-binding residues of YAP (85‒99) have been extensively modified, including the introduction of non-natural amino acids at key locations. In particular, the hydrophobic core residue of YAP, M^86^, is replaced with 6-chlorotryptophan W(6-Cl) and the L^91^ is replaced with cyclobutylalanine (Cba) (Figure 2C)—both these substitutions are crucial for improving the potency. As with other cyclic peptides, the F^95^ residue of YAP is unaltered because this residue is already ideally positioned, and it is difficult to improve binding further through modifications. To substitute the cation-π interaction, the authors used a different approach, extending the aromatics of F^96^ through replacing the phenylalanine with 1-napthyalanine.

In regard to using small molecules to occupy interface 3, we have shown that the drug flufenamic acid has this ability [13]. Partial density corresponding to the drug was observed in this region when the TEAD crystals were soaked in flufenamic acid. Flufenamate roughly occupies the region on TEAD that is occupied by two of YAP’s three interface 3 core residues, namely M^86^ and F^95^. So far this remains the only crystal structure of a small molecule that has been found to occupy interface 3. In an NMR-based fragment screen, we have also identified a benzimidazole-containing fragment that interacts with the residues near interface 3 (Table 1) [26]. As discussed below, compounds containing dioxo-benzoisothiazole or triazole-carbohydrazone scaffolds and compounds **3** and **3.1** also presumably target the interface 3 pocket on TEADs.

### 4.1. Dioxo-Benzoisothiazole Scaffold

Inventiva Pharma has identified that compounds with a dioxo-benzoisothiazole scaffold occupy the pocket on TEAD interface 3 (WO2017064277) that could be a potential PPID [27]. Both fragment-based and high-throughput screening methodologies were used to identify this TEAD-binding ligand. Chemical shift changes from NMR studies were used to verify whether the compounds occupy interface 3. Using molecular docking that was aimed to predict the binding pose of one of the Inventiva compounds (example 22) at interface 3, it was shown that this compound makes crucial hydrogen bonds as well as hydrophobic interactions with residues of interface 3 pocket (Table 1) [52]. However, when we docked example 22 and used the entire transactivation domain of TEAD, we noticed a binding pose for example 22 that occupies the interface 2 region instead. Interestingly, the compound nicely overlaps with the L^65^xxL^68^F^69^ motif of YAP: dioxo-benzoisothiazole overlays with L^65^, hydroxyethyl overlays with L^68^, and methoxyphenol overlays with the F^69^ residue. Adding these compounds to cells significantly decreased the expression of YAP/TAZ-dependent genes. Therefore, Inventiva is evaluating these compounds for treatment of malignant pleural mesothelioma where YAP/TAZ plays a prominent role in pathogenesis. However, the potency of this scaffold has been questioned because it was unable to act as an effective PPID in a fluorescence polarization (FP) assay that used a fluorescently labeled YAP peptide derived from residues that are part of interface 3 [52].

### 4.2. Triazole-Carbohydrazone Scaffold

A library of 175,000 compounds from the ZINC database that is enriched in PPIDs was virtually screened to identify TEAD-binding ligands that would occupy interface 3 [28]. Four chemical families were identified, and compounds with a triazole-carbohydrazone scaffold (hit 2) (Table 1) reduced TEAD-dependent gene expression. Hit 2 was docked to human TEAD interface 3, centered on the K289 residue of TEAD1. Interestingly, the K289 residue makes a double-pronged hydrogen bond with the carbohydrazone linker, and both the carbonyl and the imino-nitrogen of the linker interact with K289 through hydrogen bonds.

### 4.3. Compounds ***3*** and ***3.1***

Compound **3** was identified from a docking screen that virtually screened 8 million compounds to identify those that occupy the TEAD interface 3 pocket [29]. Compound **3.1**, an analog of **3**, possess similar binding attributes but is a relatively more potent PPID (Table 1). Compound **3** has been shown to disrupt YAP-TEAD interaction in co-IP assays and has also been shown to inhibit TEAD reporter activity [29].

## 5. Verteporfin Binds to YAP

Verteporfin is the probably the only PPID thus far identified that has a unique mode of action in that it binds to YAP and not TEAD [12]. Verteporfin is a benzoporphyrin derivative that was identified in a TEAD luciferase reporter screen of over 3300 drugs from the Johns Hopkins Drug library, which contains FDA-approved drugs and drugs that have entered phase 2 trials. Verteporfin can disrupt the YAP-TEAD interaction in vivo because it inhibited YAP-dependent proliferation in a transgenic mouse that overexpresses YAP and in an NF2 mutant mouse that has enhanced YAP activity [12]. Those who investigate Hippo signaling frequently use verteporfin as a pharmacological tool to disrupt YAP-TEAD interaction. However, it has limitations concerning potency, selectivity, and pharmacology, so optimization is needed. On this front, an analog of verteporfin, a divinyldipyrrine, has been identified that can inhibit TEAD-dependent transcription [53]. Its mechanism of action is not entirely clear, and whether it is a PPID remain to be determined.

## 6. Compounds That Occupy the Central Pocket

**Figure 3:**
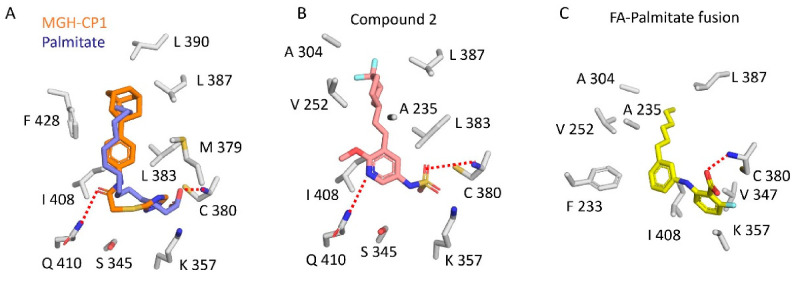
Small molecules that occupy the central pocket of TEAD2. Crystal structures showing the conformations of palmitate and MGH-CP1 (**A**), compound **2** (**B**), and FA-Palmitate fusion compound (**C**) in the central pocket of TEAD2. PDB IDs: 5HGU (Palmitate), 6CDY (MGH-CP1), 6UYC (compound **2**), 6VAH (FA-Palmitate fusion). The side chains of residues that surround these ligands are shown as grey sticks, putative hydrogen bonds are shown as dashed red lines.

We identified a novel pocket in the center of the TEAD transactivation domain that is accessible and druggable [13]. According to PockDrug, a druggability prediction server, the druggability probability for the central pocket is 1.0, in comparison, the interface 3 pocket has a score of 0.53, while interface 2 has poor druggability probability. The central pocket also offers the possibility to develop TEAD-selective compounds. The majority of residues that line this pocket are conserved among TEADs, but there are 4 residues that are non-conserved in at least one of the TEADs. These can be leveraged to design selective compounds [7]. As the central pocket has a favorable druggablility probability, there has been a marked increase in the identification of small molecules that can occupy this pocket. Although, compounds belonging to diverse chemical classes were shown to bind to this pocket, they still exhibit some common underlying features. Most of the compounds are flexible and often adopt a L-shaped conformation after binding that is also adopted by the natural ligand, palmitate (Figure 3). They have a predominant hydrophobic portion; the hydrophilic portion of the compounds faces the opening of the central pocket. This is in line with the nature of the central pocket—the bulk of the pocket is hydrophobic except for the solvent-exposed opening where the polar/charged residues are located (Figure 1C). The hydrophilic portion of a small molecule interacts with these residues by forming hydrogen bonds. Below, we describe the ligands that occupy this pocket.

### 6.1. Flufenamic Acid

We first showed that the drug flufenamic acid (FA) occupies the central pocket and inhibits TEAD activity [13]. Interestingly, TEADs are auto-palmitoylated, and a conserved, reactive cysteine residue that mediates this process has been identified [31,32]. Auto-palmitoylation of TEADs has been shown to be important for TEAD stability and activity and potentiates TEAD interaction with YAP. Auto-palmitoylation is also regulated by cell density [54]. Although, we identified that FA also occupies the interface 3 site, we believe that the central pocket is the primary binding site for FA. The TEAD-FA crystal structure showed that the ligand has an occupancy of 1.0 at this site and makes extensive hydrophobic interactions with the residues lining the central pocket. Moreover, a hydrogen bond exists between the FA carboxyl group and the main chain amide group of the reactive cysteine. Our subsequent NMR-based analysis indicates that some of the residues lining the central pocket undergo chemical shifts upon FA addition, whereas no significant changes to TEAD interface 3 residues were observed [26]. FA is not a PPID, but when added at higher concentrations, it can inhibit TEAD palmitoylation in vitro [26]. FA perhaps inhibits TEAD activity by acting as a palmitoylation inhibitor rather than acting as an allosteric PPID. Inhibiting TEAD palmitoylation alone without the disruption of its interaction with YAP/TAZ has also emerged as a strategy to inhibit TEAD activity; compound **2** and the vinylsulfonamides, as discussed below, belong to this class.

Interestingly, a chloromethyl ketone analog of FA, TED-347, can covalently modify the reactive cysteine and act as an allosteric PPID [33]. Although, the density of TED-347 in the crystal structure is not that convincing, molecular modeling indicated that covalent bond formation is a key determinant for disrupting the YAP-TEAD interaction. In agreement with this study, modifying FA with an acrylamide warhead as in the compound MYF-01-037 (Table 2) enables it to covalently modify the reactive cysteine and allows it to function as an allosteric PPID [34]. The disruptive ability of MYF-01-037 was assessed using a split luciferase assay that reports YAP-TEAD interaction through an increase in luminescence. The compound is also able to inhibit the expression of connective tissue growth factor (CTGF), a YAP target gene. Molecular docking suggested that the compound occupies the central pocket and is covalently bonded to the cysteine in TEAD that is otherwise palmitoylated. The authors also confirm on-target engagement of the compound in cells by showing that a biotinylated version can directly pull down TEAD from whole-cell lysates, but when cells were treated with non-biotinylated MYF-01-037, the amount of TEAD in the pull down was strikingly reduced.

Given their chemical similarity to FA, a set of non-fused tricyclic compounds from Vivace Therapeutics [35] (WO2018204532) are also expected to bind to the central pocket (Table 2). However, with a lack of structural information, it is difficult to predict the exact binding mode. Vivace synthesized over 200 analogs and evaluated their potency using a TEAD reporter assay. Potent compounds were further investigated in a tumor suppression assay using an immuno-compromised mouse model, and the compounds were also evaluated for their ability to inhibit cell proliferation. The mechanism of action of these small molecules is not fully known, and it is not clear whether they act as PPIDs. Vivace patented three variants of the non-fused tricyclic compounds: (i) Benzosulfonyl compounds where a sulfonamide substituent is introduced in the non-fused tricyclic scaffold (WO2019040380), (ii) oxadiazole compounds where the tetrazole is replaced with an oxadiazole (WO2019222431), and (iii) benzocarbonyl compounds where a carbonyl substituent was introduced in the non-fused tricyclic scaffold (WO2019113236).

Recently, through the efforts of the Structural Genomics Consortium, an FA analog that appears to be a fusion of FA and palmitate has been identified (RCSB Protein Data Bank ID: 6VAH (Figure 3; Table 2). The trifluoro substituent of FA is replaced by a heptyl group, and the crystal structure showed that the heptyl substituent extends deep into the central pocket and makes further extensive hydrophobic interactions. Therefore, we predict that this fusion would have better potency than FA.

### 6.2. Non-Covalent Allosteric PPIDs

#### 6.2.1. MGH-CP1

A library of 50,000 compounds were screened in a TEAD reporter assay, and hits were tested for their ability to inhibit TEAD palmitoylation *in vitro* (WO2017053706). As a first example, one of the compounds, MGH-CP1, remarkably inhibited palmitoylation of endogenous TEAD in cells [36]. This suggests that TEAD palmitoylation is a dynamic event and small molecules could be used to interfere with this activity. MGH-CP1 is also the first example of an allosteric PPID because results of co-immunoprecipitation assays suggested that it disrupted the YAP-TEAD interaction. Although, the crystal structure of the TEAD with MGH-CP1 showed that it occupies the central pocket, it did not alter the overall conformation of the TEAD transactivation domain, so the YAP/TAZ-binding surface pockets were intact. Therefore, it is unclear how MGH-CP1 is able to act as a PPID. As co-immunoprecipitation experiments used full-length proteins, it is possible that MGH-CP1 could force full-length TEAD to adopt a conformation that is unfavorable for YAP interaction.

In the central pocket, MGH-CP1 adopts a L-shaped conformation similar to that of palmitate (Figure 3A). It interacts with TEAD primarily through hydrophobic contacts. The molecule is oriented such that the triazole is close to the opening of the central pocket, near the reactive cysteine, whereas the adamantane is buried deep in the pocket. The carbonyl group in the linker makes a hydrogen bond with a side chain of glutamine (Q410, TEAD2) (Figure 3A). The compound, however, had poor pharmacokinetics properties as it was cleared quickly when orally administered to mice, but it was effective enough to inhibit TEAD activity in the intestinal epithelium. Using MGH-CP1 as a pharmacological tool, the authors showed that TEAD activity is dispensable for normal intestinal homeostasis.

#### 6.2.2. DNA-Encoded Indole-Focused Ugi-Peptidomimetics

A DNA-encoded, indole-focused combinatorial library of 8112 compounds was screened against TEAD with the goal of identifying PPIDs that disrupt the YAP-TEAD interaction [37]. The indole-focused library is expected to exploit the crucial PPI interactions mediated by the indole ring of the tryptophan residues in proteins. A crucial tryptophan is located at the TEAD interface 3 and the TEAD central pocket also accommodates tryptophan-like aromatic residues. The authors identified a small molecule allosteric PPID (Compound **9**) that can occupy the central pocket and disrupt YAP-TEAD interaction (Table 2). Derivatives of this compound were also made to improve potency. In order to validate that it occupies the central pocket, a palmitate-based FP assay was employed, and compound 9 effectively prevented the interaction of TEAD with palmitate. This compound was also able to disrupt the interaction between YAP and TEAD in an in vitro FP assay using a YAP peptide, thereby acting as an allosteric PPID. As only the transactivation domain of TEAD was used in this assay, it will be interesting to deduce whether the compound induces a conformational change in TEAD upon binding. Unfortunately, in HEK 293 cells, compound **9** did not inhibit the expression of CTGF, a YAP-TEAD target gene [37]. Intriguingly, the efficacy is better when compound **9** is combined with a hippo kinase inhibitor.

#### 6.2.3. Dihydropyrazolo Pyrimidines

Compounds with a dihydropyrazolo pyrimidine scaffold have been shown to function effectively as disruptors in the nanomolar range in an *in vitro* TR-FRET assay that monitors the interaction between the TEAD transactivation domain and YAP peptide (WO2019232216) [38]. Transactivation domains of TEAD2 and TEAD3 were used, and compounds that bind specifically to either of the TEADs as well as compounds that bind equally well to both TEAD2 and TEAD3 were identified. However, it is unclear whether they bind to the surface of TEADs or the central pocket. When we performed molecular docking using AutoDockVina, the results suggest that they occupy the central pocket (data not shown). Consistently, these molecules also possess the characteristics of a central pocket binding ligand (Table 2). They are flexible enough to adopt a palmitate-like conformation and have a predominant hydrophobic portion that buries deep into the central pocket and a small hydrophilic portion that is solvent-exposed. The ability to develop TEAD2- and TEAD3- selective compounds also supports the hypothesis that the compounds occupy the central pocket as they may exploit the non-conserved residues of the central pocket to impart selectivity.

### 6.3. Allosteric PPIDs That Form a Covalent Interaction

All TEADs have a conserved reactive cysteine in the central pocket that is palmitoylated. Other than TED-347 and MYF-01-037, few compounds have been identified that bind to the central pocket are able to exploit the reactivity of this cysteine by forming a covalent bond.

#### 6.3.1. K-975

The covalent allosteric PPID K-975 (Table 2) also has an acrylamide warhead and has been shown to inhibit TEAD palmitoylation as well as disrupt YAP/TAZ-TEAD interaction [39]. The crystal structure of the compound bound to TEAD showed that it occupies the central pocket, and co-immunoprecipitation studies indicated that it can disrupt the YAP/TAZ-TEAD interaction. Its anti-tumorigenic properties were verified in both malignant pleural mesothelioma cell lines and xenograft models. K-975 did display renal toxicity therefore a better analog is needed for its clinical use as a disruptor.

#### 6.3.2. Kojic Acid Analogs

In an attempt to identify small molecules that would occupy the central pocket of TEAD, a new fluorescence-based screen was developed that used CPM (*N*-(4-(7-diethylamino-4-methylcoumarin-3-yl)phenyl)maleimide, a thiol-reactive fluorescent probe [40]. CPM fluorescence increased when forming a thiol conjugate with the reactive cysteine. Kojic acid analogs were identified as compounds that inhibit CPM fluorescence, suggesting that they occupy the central pocket. This was further verified using NMR-based ^1^H-^15^N-HSQC analysis. Various studies using kojic acid analogs, mutant TEADs, and mass spectrometry analysis revealed that kojic acid analogs covalently modify the central pocket reactive cysteine. Through in vitro thermal shift analysis, kojic acid has been shown to destabilize TEAD. By destabilizing or altering the conformation of TEAD, it is perhaps able to prevent YAP from interacting with TEAD and act as an allosteric PPID.

### 6.4. Other Central Pocket Binders

Small molecules have been identified that occupy the TEAD central pocket, but they do not act as allosteric PPIDs; rather, they act either as TEAD palmitoylation inhibitors or activators.

#### 6.4.1. Compound **2**

Using a fluorescence polarization screen with BODIPY-conjugated palmitate as a tracer, 1 million compounds were screened, and compound **1** was identified as a chemical matter that interferes with palmitate binding to TEAD [41]. However, it did not disrupt the YAP-TEAD interaction. Compound **2**, a more potent analog of compound **1**, was crystallized in complex with TEAD2. The structure showed that it occupies the central pocket (Figure 3B and Table 2). The sulfonamide in this compound is solvent-exposed and also makes a hydrogen bond with the main chain of cysteine that is otherwise palmitoylated (Figure 3B). The pyridine next to the sulfonamide in compound **2** forms a hydrogen bond with a glutamine side chain (Q410). These bonds are reminiscent of the hydrogen bonds formed by compounds that were previously shown to occupy the central pocket: the carboxyl group of FA forms a hydrogen bond with the main chain of cysteine and the carbonyl group of MGH-CP1 forms a hydrogen bond with glutamine. Compound **2** is able to occupy the central pocket and stabilize TEAD in vitro, similar to palmitate. However, in vivo addition of this compound enables TEAD to act as a transcriptional repressor, and TEAD behaves in a dominant-negative fashion primarily through reducing its chromatin occupancy. Adding compound **2** to JHH-7 cells reduces the expression of YAP/TAZ-dependent genes and compound **2** reduces tumor growth in a xenograft model [41].

#### 6.4.2. Vinylsulfonamides

Like acrylamide warheads, vinylsulfonamide warheads have also been successfully employed to target the reactive cysteine in TEADs. Vinylsulfonamides were identified through a virtual screen that predicted that they will occupy the central pocket. Consistently, they have been shown to prevent TEAD palmitoylation [42]. Vinylsulfonamide analogs do not disrupt the YAP-TEAD interaction; however, interfering with TEAD palmitoylation appears to be sufficient to inhibit TEAD activity. The most potent and selective vinylsulfonamide analog is the DC-TEADin02 (Table 2), which has an IC_50_ of 200 nM. It also displays selectivity for TEAD over other kinase and lipid-binding proteins.

#### 6.4.3. Quinolinols

Compounds that occupy the central pocket inhibit TEAD activity either by allosterically disrupting the YAP-TEAD interaction or by inhibiting TEAD palmitoylation. However, quinolinols have the unique ability to stimulate TEAD activity [43]. We identified quinolinols as compounds that occupy the central pocket using a target-based TEAD-palmitate AlphaScreen. Surface plasmon resonance measurements indicated that they bind to TEAD in the low micromolar range [43]. Docking and molecular dynamics simulation show that quinolinols stably occupy the central pocket, and obstructing the TEAD pocket using a bulky amino acid reduced the quinolinol binding affinity for TEAD. The YAP/TAZ-TEAD signature is enriched after treating cells with quinolinols. Therefore, quinolinols possess palmitate-like TEAD stimulating activity. This activity was exploited to improve cutaneous wound healing in mouse, which is in line with previous observations that used genetic methods to show that YAP/TAZ activity improves wound healing [55,56].

## 7. Conclusions and Future Perspectives

The last five years have been an exciting time for those seeking ways to target and disrupt the YAP-TEAD complex. This complex has served as a model for innovative PPID development. Elegant peptidomimetic approaches through the introduction of non-natural amino acids and disulfide linkages greatly improved the potency of synthetic peptides over wild-type YAP. Although a difficult target, small molecules that exploit the TEAD surface pockets have also been identified. Yet none of the molecules have been convincingly shown to act as PPIDs in cells. The limitation largely stems from the nature of the surface pockets that makes it difficult to design high affinity small molecules to effectively disrupt the YAP/TAZ-TEAD interaction. Peptidomimetics are able to overcome this problem as they are able to bind to TEAD potently, but they have difficulty in penetrating cells. To disrupt this high affinity nuclear complex both cellular penetration and potency are crucial, therefore the properties of small molecules and peptidomimetics have to be combined for an effective surface-targeting PPID. The central pocket is an ideal choice for small molecule approaches. Although, it is expected to find small molecules that compete with palmitate and act as TEAD palmitoylation inhibitors, it is surprising to see that some palmitoylation inhibitors also have the ability to allosterically disrupt the formation of the YAP-TEAD complex. The central pocket has the features that allow the development of potent small molecules. Additionally, selective TEAD inhibitors can potentially be developed by exploiting the non-conserved residues of the central pocket. However, the biological need to develop such compounds is not established. In the near future, we expect design of more innovative molecules, e.g., molecules that target both the surface and central pockets, peptide-small molecule conjugates and PROTACs that degrade TEADs. We look forward to seeing a first-in-class agent that disrupts the YAP/TAZ-TEAD complex and displays efficacy in an oncology clinical trial.

## Figures and Tables

**Table 1:** Compounds that target TEAD surface pocket interfaces 2 and 3.

No.	Molecule	Structure	Surface Pocket Validation	Molecule Type	Validation Method	Reference
Modalities binding to Interface 2						
1.	TB2G1	Cystine-dense peptide	Rosetta modeling	PPID	Co-IP assay	[21]
2.	Fragment 1	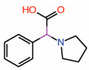	Crystal structure	PPID unlikely	GST pull-down	[22]
3.	Tri-substitutedpyrazoles	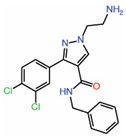	Crystal structure	Potential PPID		
Modalities binding to Interface 3						
4.	Peptide 17	YAP cyclic peptide	Molecular modeling	PPID	GST pull-down	[23]
5.	Peptide 10	YAP cyclic peptide	Crystal structure	PPID	GST pull-down	[24]
6.	Peptides 9, 10	YAP linear peptide	Crystal structure	PPID	TR-FRET assay	[25]
7.	Flufenamic acid	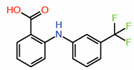	Crystal structure	PPID unlikely		[13]
8.	TEAD-binding fragment	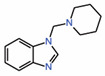	NMR	PPID unlikely		[26]
9.	Dioxo-benzoisothiazole Example 22	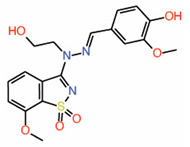	NMR	PPID	AlphaLISA assay	[27]
10.	Triazole carbohydrazides Hit 2	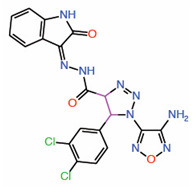	Molecular modeling	Potential PPID		[28]
11.	Compound **3.1**	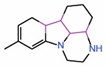	Molecular docking	PPID	Co-IP assay	[29]
Peptide binding to both Interfaces 2 & 3						
12.	Super-TDU	YAP-VGLL4 fusion peptide	Molecular modeling	PPID	Co-IP assay	[30]

**Table 2:** Compounds that target the TEAD central pocket.

No.	Molecule	Structure	Binding Validation	Molecule Type	Validation Method	Reference
Flufenamates						
1.	Flufenamic acid	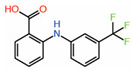	Crystal structure	Palmitoylation inhibitor	Mass Spec analysis	[13]
2.	TED-347	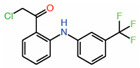	Crystal structure	Allosteric PPID	FP assay with YAP peptide	[33]
3.	MYF-01-037	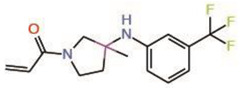	Molecular docking	Allosteric PPID	Split luciferase assay	[34]
4.	Non-fused tricyclic Compound **42**	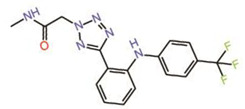	unknown	PPID?		[35]
5.	FA-Palmitate fusion	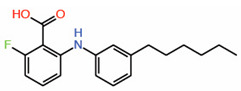	Crystal structure	PPID?		
Non-covalent allosteric PPIDs						
6.	MGH-CP1	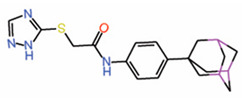	Crystal structure	Allosteric PPID	Co-IP assay	[36]
7.	Compound **9** Indole-focused	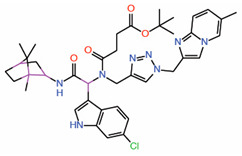	Palmitate-based FP assay	Allosteric PPID	FP assay with YAP peptide	[37]
8.	Dihydropyrazolo pyrimidines Compound **7**	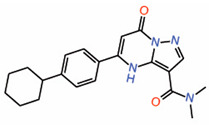	unknown	AllostericPPID?	TR-FRET assay	[38]
Covalent allosteric PPIDs						
9.	K-975	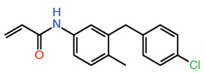	Crystal structure	Allosteric PPID	Co-IP assay	[39]
10.	Kojic acid analogs Compound **19**	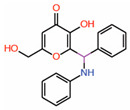	Thiol conjugation assay	Allosteric PPID	FP assay with YAP peptide	[40]
Other central pocket binders						
11.	Compound **2**	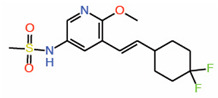	Crystal structure	Palmitoylation inhibitor	FP assay with palmitate	[41]
12.	Vinylsulfonamide DC-TEADin02	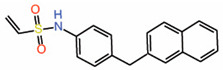	Molecular docking	Palmitoylation inhibitor	Competitive NMR	[42]
13.	Quinolinol Q2	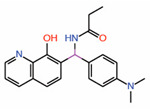	Molecular dynamics simulations	TEAD activator	RNA-Seq	[43]
